# A New Entropy-Based Atrial Fibrillation Detection Method for Scanning Wearable ECG Recordings

**DOI:** 10.3390/e20120904

**Published:** 2018-11-26

**Authors:** Lina Zhao, Chengyu Liu, Shoushui Wei, Qin Shen, Fan Zhou, Jianqing Li

**Affiliations:** 1 School of Control Science and Engineering, Shandong University, Jinan 250061, China; 2 School of Instrument Science and Engineering, Southeast University, Nanjing 210096, China; 3 Department of Cardiovascular Medicine, First Affiliated Hospital of Nanjing Medical University, Nanjing 210036, China

**Keywords:** atrial fibrillation (AF), sample entropy (SampEn), fuzzy measure entropy (FuzzyMEn), coefficient of sample entropy (COSEn), wearable ECG, RR time series, cardiac rhythm, 87.19.Hh, 87.19.ug, 87.19.uj, 87.85.Ng, 05.45.Tp

## Abstract

Entropy-based atrial fibrillation (AF) detectors have been applied for short-term electrocardiogram (ECG) analysis. However, existing methods suffer from several limitations. To enhance the performance of entropy-based AF detectors, we have developed a new entropy measure, named Entropy_AF_, which includes the following improvements: (1) use of a ranged function rather than the Chebyshev function to define vector distance, (2) use of a fuzzy function to determine vector similarity, (3) replacement of the probability estimation with density estimation for entropy calculation, (4) use of a flexible distance threshold parameter, and (5) use of adjusted entropy results for the heart rate effect. Entropy_AF_ was trained using the MIT-BIH Atrial Fibrillation (AF) database, and tested on the clinical wearable long-term AF recordings. Three previous entropy-based AF detectors were used for comparison: sample entropy (SampEn), fuzzy measure entropy (FuzzyMEn) and coefficient of sample entropy (COSEn). For classifying AF and non-AF rhythms in the MIT-BIH AF database, Entropy_AF_ achieved the highest area under receiver operating characteristic curve (AUC) values of 98.15% when using a 30-beat time window, which was higher than COSEn with AUC of 91.86%. SampEn and FuzzyMEn resulted in much lower AUCs of 74.68% and 79.24% respectively. For classifying AF and non-AF rhythms in the clinical wearable AF database, Entropy_AF_ also generated the largest values of Youden index (77.94%), sensitivity (92.77%), specificity (85.17%), accuracy (87.10%), positive predictivity (68.09%) and negative predictivity (97.18%). COSEn had the second-best accuracy of 78.63%, followed by an accuracy of 65.08% in FuzzyMEn and an accuracy of 59.91% in SampEn. The new proposed Entropy_AF_ also generated highest classification accuracy when using a 12-beat time window. In addition, the results from time cost analysis verified the efficiency of the new Entropy_AF_. This study showed the better discrimination ability for identifying AF when using Entropy_AF_ method, indicating that it would be useful for the practical clinical wearable AF scanning.

## 1. Introduction

Atrial fibrillation (AF) is a typical arrythmia, defined as a tachyarrhythmia characterized by predominantly uncoordinated atrial activation with consequent deterioration of atrial mechanical function [1,2]. AF is associated with significant mortality and morbidity, resulting in that more than 12 million Europeans and North Americans suffer from AF [3,4]. Moreover, its prevalence increases with age, from <0.5% at 40–50 years of age, to 5–15% for 80 year olds [5]. However, current diagnose for AF is under-detected and under-diagnosed due to the asymptomatic characteristic of AF episode, which can elude clinical detection [6]. Timely and accurate detection of AF for real-time monitoring and feedback is therefore challenging [7,8].

Twelve-lead 24-h Holter is a common method in clinic for AF detection. This technique is effective to diagnose patients suffering from persistent AF but may miss many cases of paroxysmal AF [9]. Wearable and longer-term electrocardiogram (ECG) monitoring strategies, especially the techniques with the characteristics of unobtrusive, convenient and inexpensive, would contribute to prevention of AF-itself and AF-related complications, including stroke and heart failure [6,8]. Wearable and long-term ECG monitoring requires more robust AF detector. Traditionally, atrial activity analysis-based [10] and ventricular response analysis-based methods are two common approaches for AF analysis. Compared with the former, the latter is more suitable for wearable monitoring since the detection of the absence of P waves in dynamic ECGs for atrial activity analysis is difficult and even impossible [3]. In contrast, ventricular response analysis only use the RR interval information derived from the most obvious amplitude feature of QRS complexes [11]. In the past decade, many ventricular response analysis-based AF detectors have developed [8,11,12,13,14]. Park et al. proposed a Poincare plot method using the inter-beat intervals and extracted three features form the Poincare plot to classify AF and non-AF rhythms (sensitivity 91.4% and specificity 92.9%) [15]. Sarkar et al. proposed a Lorenz plot method for continuous long-term AF monitoring for an implantable monitor device [16]. Tateno and Glass proposed a method using the information of coefficient of variation and density histograms of RR and deltaRR intervals [17]. Linker filed a US patent describing the use of a median absolute deviation (MAD) method for AF detection [18]. Garcia et al. used a novel method exploiting the relative wavelet energy (RWE) and signal averaging technique to automatically detect AF episodes of a wide variety in length [19]. Petrėnas et al. developed AF detector using the characteristics of RR interval irregularity and noise level [20]. Researchers also developed various entropy methods, which have been proven the potential in analyzing short time window of RR time series and generating a timely feedback of AF diagnosis [12,13,21].

Entropy refers to the degree of regularity or irregularity of a time series and is estimated by counting how many ‘template’ patterns repeat. AF is typically presented by the irregular RR time series and the irregularity can be captured by the entropy. Richman et al. [22] developed sample entropy (SampEn) in 2000, which has been the most widely used entropy methods for short-term physiological signal analysis and it also has applications in AF detection. A benefit of SampEn is its ability to use short runs or bursts of AF as a template for matching, hence avoiding issues relating to short AF episode durations that are common with RR interval variability-based methods [8]. Previous studies showed that SampEn provided a high degree of accuracy in distinguishing AF from sinus rhythm, but encountered errors when atrial or ventricular ectopy were present [11,23]. Since SampEn does not count self-matches and lowers the counts of vector matching, it can result in infinite or indeterminate outputs and is especially problematic for short-term time series [23]. Lake and colleagues developed a new entropy-based AF detector named coefficient of sample entropy (COSEn), with two key improvements from SampEn: flexibility in choosing the distance threshold r, and adjusting for heart rate by subtracting the negative natural log of the mean RR interval. COSEn reported an area under curve (AUC) of 92.8% when testing on the MIT-BIH Atrial Fibrillation (MIT-BIH AF) database and also achieved positive predictive values higher than 90% when used on the University of Virginia Holter database [23]. Liu et al. previously found that weak statistical stability existed in SampEn due to the rigid similarity rule of 0–1 determination [24], and thus developed a fuzzy measure entropy (FuzzyMEn) by employing the fuzzy function to replace the 0–1 Heaviside function [25]. Then they proposed a normalized fuzzy entropy-based AF detector (NFEn), that combines the advantages of both COSEn and FuzzyMEn.

No matter whether SampEn, or FuzzyMEn, or COSEn, even or NFEn, they all use Chebyshev distance to quantify the similarity of two state vectors, i.e., only considering maximum difference between two state vectors. However, Chebyshev distance has two limitations [26]. First, it is not normalized as it has no upper limit and can lead to an unbounded range for the tolerance threshold *r* in the probability estimation. Second, only considering the maximum difference between two state vectors is blind to the range of their element-wise difference, i.e., the element differences except the maximum one could not be quantified. Thus, Omidvarnia et al. modified the SampEn by re-defining the distance function using ranged function to replace Chebyshev function and thus proposed a new range entropy (RangeEn) [26]. RangeEn showed more robust to signal amplitude changes and more sensitive to in-built self-similarity of time series than SampEn, suggesting it is a good candidate for studying complex physiological time series.

In this study, we sought to combine the concepts of the ranged function for distance determination and the normalized fuzzy entropy AF detector—NFEn, and to develop a new entropy-based AF detection method. The new method involves the five improvements:

(1) uses a ranged function to define the vector distance as RangeEn did,
(2) uses a fuzzy function rather than the Heaviside function to determine vector state similarity,
(3) replaces probability estimation with density estimation for entropy calculation,
(4) utilizes a flexible distance threshold *r*, and
(5) adjusts for heart rate by subtracting the natural log value of the mean RR interval.

We evaluated the new method using both the online open-access MIT-BIH AF database and the actual clinical wearable long-term ECG recordings from AF patients. The clinical ECG data were collected using a wearable SmartVest-based ECG recording device, which offers a continuous, low-cost, unobtrusive, and convenient solution for long-term ECG monitoring. The objective was 2-fold: (1) to determine the performance of the new method for AF detection in a population of patients with known AF (online open-access MIT-BIH AF database) and (2) to assess the AF detection accuracy among actual clinical patients with AF.

## 2. Methods

### 2.1. Data

The AF data used in this study included both the online open-access database and the AF recordings collected from clinic using a wearable device.

#### 2.1.1. Online Open-Access Database

The MIT-BIH AF database was used and it included 25 long-term ECG recordings, with the detailed QRS position and beat annotation files. The individual ECG recordings were each 10 h in duration and were sampled at 250 Hz, giving a minimum time resolution of 4 ms for RR time series [3,12]. For all recordings, the rhythm annotations were prepared manually for four types: AF (atrial fibrillation), AFL (atrial flutter), J (AV junctional rhythm), and N (used to indicate all other rhythms). The types of AF (42.6%) and N (54.2%) are the majority (see Table 1). RR episodes corresponding to the four rhythm types (AF, AFL, J and N) were extracted. Then data pre-processing was performed on the classified RR episodes. A 30-beat window length was used to segment the RR episodes without overlap. Classification was firstly performed between AF and N rhythm types. Then, the RR segments corresponding to the N, AFL and J types were merged as non-AF rhythms to enable classification between AF and non-AF rhythm types. Table 1 shows the detailed database profile, as well as the numbers of RR segments after the segmenting procedures.

#### 2.1.2. Clinical Wearable AF Recordings

Ten AF patients were included in this study (six females and four males, aged from 31 to 64). All of the patients were recruited from the First Affiliated Hospital of Nanjing Medical University, Jiangsu, China, and they were diagnosed as AF through the ECG Holter test. The protocol of this study was approved by the Ethics Committee of the First Affiliated Hospital of Nanjing Medical University. All patients gave written informed consent. Long-term (24 h) ECG data during patients’ daily activities were collected using our developed Wearable ECG SmartVest System with a sample rate of 500 Hz and a 12-bit resolution [27]. Signal recording lasted from May 2018 to July 2018. The wearable ECG SmartVest System can provide an Internet of Things (IoT)-driven 24/7 ECG monitoring for patients, and it consists of four typical IoT components, i.e., sensing layer, network layer, cloud platform and application layer. The limb Lead-II ECG was selected for the following analysis since this lead usually has the maximum signal amplitude and can facilitate the QRS detection. 

First, signal quality assessment was performed for the wearable ECGs as a preprocessing stage to filter the noisy episodes, which can significantly influence the accurate QRS locations. Five signal quality indices (SQIs) were used, including bSQI, tSQI, iSQI, pSQI and kSQI. Table 2 lists the SQIs with the descriptions. The detailed definitions can refer to [27]. A 10-s ECG was determined as noisy if two of the following criteria meet: (1) bSQI < 0.5, (2) tSQI = 0, (3) iSQI = 0, (4) pSQI < 0.8, (5) kSQI < 3. For the 10 24-h ECG recordings, 31.6% were identified as noisy signals and were excluded the following AF detection (see Table 3).

After the signal quality assessment, a lightweight QRS detector was performed on the ECG episodes with good signal quality for locating QRS complexes [27]. Ectopic RR intervals were identified and excluded using our previously developed combined method [30]. A 30-beat window length was also used to segment the RR interval time series without overlap. Three clinical staffs visually inspected each 30-beat RR segment as AF or non-AF rhythm, and this work was done independently with the three staffs. If they did not give the same answer, the majority decision rule was performed. Table 3 shows the detailed clinical database profile.

### 2.2. New Entropy-Based AF Detection Method

The conception of the new entropy-based AF detection method came from the following aspects. First, entropy measures the conditional probability that two short vectors of length m that match within a distance tolerance r will also match at the m+1 st point. Thus, the determination for vector similarity is a core step, and it relays on the measure of the distance between two vectors. Traditionally, Chebyshev distance (i.e., the element maximum distance) is used but it lacks the upper boundary limit. Herein, we normalized the distance between two vectors using ranged function as refer to Omidvarnia et al.’s work [26]. Second, once we have the distances between the two vectors, we can determine their similarity or dissimilarity using a determination rule function. Traditionally, Heaviside function is used to classify vector similarity in a binary fashion. This rigid determination results in weak statistical stability [25]. Here we use a fuzzy function to replace the Heaviside function to smooth the decision boundary and to reduce the sensitivity of entropy outputs to small changes in *r*. Third, the traditional SampEn method uses a probability-based estimation, which may generate bias for short RR interval time series. Lake et al. suggested replacing probability estimate with density estimate for entropy approximation and demonstrated the improved version of COSEn has better performance than SampEn for AF detection [23]. Herein, we also employ a density-based estimation to generate the AF entropy value. Fourth, setting an appropriate tolerate threshold *r* is important for entropy method and herein we utilize a flexible threshold *r*, which uses a minimum average matching number for vector similarity. Last but not least, since AF is usually accompanied by a quick heart rate, the adjustment for heart rate is necessary for the AF entropy calculation. Herein, we adjust for heart rate by subtracting the natural log value of the mean RR interval for entropy value calculation. We summarize the calculation process for the new AF entropy method as follows.

For an RR time series x(i) (1≤i≤N), firstly form the vector sequences $X_{i}^{m}$ (1≤i≤N−m):

(1) $$\;X_{i}^{m}\;=\;\left\{x\left(i\right),x\left(i+1\right),\cdots ,x\left(i+m-1\right)\right\}\;$$

where the vector $X_{i}^{m}$ represents m consecutive x(i).

The distance between vector sequences $X_{i}^{m}$ and $X_{j}^{m}$ doesn’t use the maximum distance (Chebyshev distance). We normalize and define the distance as:

(2) $$dX_{i,j}^{m}\;=\;d\left[X_{i}^{m},X_{j}^{m}\right]=\frac{\underset{0\leq k\leq m-1}{\max}\left|x\left(i+k\right)-x\left(j+k\right)\right|-\underset{0\leq k\leq m-1}{\min}\left|x\left(i+k\right)-x\left(j+k\right)\right|}{\underset{0\leq k\leq m-1}{\max}\left|x\left(i+k\right)-x\left(j+k\right)\right|+\underset{0\leq k\leq m-1}{\min}\left|x\left(i+k\right)-x\left(j+k\right)\right|+\varepsilon }$$

where ε is a small positive number to avoid the possible denominator of 0. Then we calculate the similarity degree $DX_{i,j}^{m}\left(n,r\right)$ between the vectors $X_{i}^{m}$ and $X_{j}^{m}$ by a fuzzy function $uX\left(dX_{i,j}^{m},n,r\right)$ defined as:

(3) $$\;DX_{i,j}^{m}\left(n,r\right)\;=\;uX\left(dX_{i,j}^{m},n,r\right)\;=\;\exp\left(-\frac{{\left(dX_{i,j}^{m}\right)}^{n}}{r}\right)\;$$

where n is the similarity weight and  r is the flexible tolerance threshold. The determination for the flexible threshold *r* is to vary *r* value from an initial value of 0.05 until a specified number of average matches for vector similarity is attained.

Then we define the functions $BX^{m}\left(n,r\right)$ as:

(4) $$\;BX^{m}\left(n,r\right)\;=\;\frac{1}{N-m}\overset{N-m}{\underset{i\;=\;1}{\sum }}\left(\frac{1}{N-m}\overset{N-m}{\underset{j\;=\;1}{\sum }}DX_{i,j}^{m}\left(n,r\right)\right)\;$$

$BX^{m}\left(n,r\right)$ measures the mean similarity degrees for the vectors at dimension m. Similarly, we define the functions of mean similarity degrees $AX^{m+1}\left(n,r\right)$ for dimension m+1 as:

(5) $$\;AX^{m+1}\left(n,r\right)\;=\;\frac{1}{N-m}\overset{N-m}{\underset{i\;=\;1}{\sum }}\left(\frac{1}{N-m}\overset{N-m}{\underset{j\;=\;1}{\sum }}DX_{i,j}^{m+1}\left(n,r\right)\right)\;$$

Then, we use a density-based estimation, rather than probability-based estimation, to generate a quadratic fuzzy entropy using the volume of each matching region, i.e., ${\left(2r\right)}^{m}$ as:

(6) $$Entropy_{AF}\;=\;-\ln\left(\frac{AX^{m+1}\left(n,r\right)/{\left(2r\right)}^{m+1}}{BX^{m}\left(n,r\right)/{\left(2r\right)}^{m}}\right)\;=\;-\ln\left(\frac{AX^{m+1}\left(n,r\right)}{BX^{m}\left(n,r\right)}\right)+\ln\left(2r\right)\;$$

We also subtract the natural log of mean RR interval as follows:

(7) $$\;Entropy_{AF}\;=\;-\ln\left(\frac{AX^{m+1}\left(n,r\right)}{BX^{m}\left(n,r\right)}\right)+ln\left(2r\right)-\ln\left(RR_{mean}\right)\;$$

where $RR_{mean}$ is the mean of RR intervals in the current RR segment. $RR_{mean}$ is expressed in unit of *s*.

As shown in Equation (7), directly subtracting the item of $\ln\left(RR_{mean}\right)$ is arbitrary. Last, we use a weight to optimized the effect of mean RR interval on the final entropy output of *Entropy_AF_* as:

(8) $$\;Entropy_{AF}\;=\;-\ln\left(\frac{AX^{m+1}\left(n,r\right)}{BX^{m}\left(n,r\right)}\right)+ln\left(2r\right)-w\times \ln\left(RR_{mean}\right)\;$$

where w is a weight for optimization.

### 2.3. Evaluation Method

SampEn, FuzzyMEn and COSEn were taken as “comparable algorithms” in this study. *Entropy_AF_*, as well as three comparable entropy measures, were calculated for each 30-beat RR segment. The four entropy measures were compared between the AF and non-AF rhythm types for both MIT-BIH AF database and clinical wearable AF database, as well as between the AF and N rhythm types for the MIT-BIH AF database. Entropy values on one side of a threshold c were labelled as AF rhythm and values on the other side of c were labelled as N or non-AF rhythm. Classifier accuracy was assessed via the following performance metrics:

- Sensitivity: Se = TP/(TP+FN)
- Specificity: Sp = TN/(TN+FP)
- Accuracy: Acc = (TP+TN)/(TP+FP+FN+TN)
- Positive predictive value: PPV = TP/(TP+FP)
- Negative predictive value: NPV = TN/(TN+FN)
- Total error: Err = (FP+FN)/(TP+FP+FN+TN)

where TP, TN, FP and FN are the numbers of true positives, true negatives, false positives and false negatives respectively.

The receiving operator curve (ROC) curve was used to evaluate the effectiveness of each entropy measure in AF classification. The ROC curve is a plot of (Se) versus (1−Sp) for many possible values of c, which varied from the minimum to the maximum of the entropy outputs, with a step of 1% of the range. AUC was used to evaluate the performances of different entropy measures. The Youden index (J), another metric for assessing ROC curves, was also calculated as:

(9) $$\;J\;=\;\underset{c}{\max}\left\{Se\left(c\right)+Sp\left(c\right)-1\right\}\;$$

At the optimal cut-point $c^{*}$, J is maximized and the classifier equally weighs sensitivity and specificity. In this study, $c^{*}$ values were determined from the MIT-BIH AF database, and used for testing on the clinical wearable AF database. The afore-mentioned performance metrics of Se, Sp, Acc, PPV, NPV and Err were given at the point of $c^{*}$. Doctors are concerned about the index’s effectiveness for accepting or excluding AF judgment with high probability. Thus, we also calculated the performance metrics at the setting of cut-point c for Se>99% and Sp>99%, respectively.

In addition, we tested the classification performance of the four entropy measures when using a lower time window, i.e., 12-beat time window. The time costs of the four entropy measures were also tested, for 30-beat and 12-beat time window, respectively.

## 3. Results

### 3.1. Results on the MIT-BIH AF Database (30-Beat Time Window)

**Figure 1:**
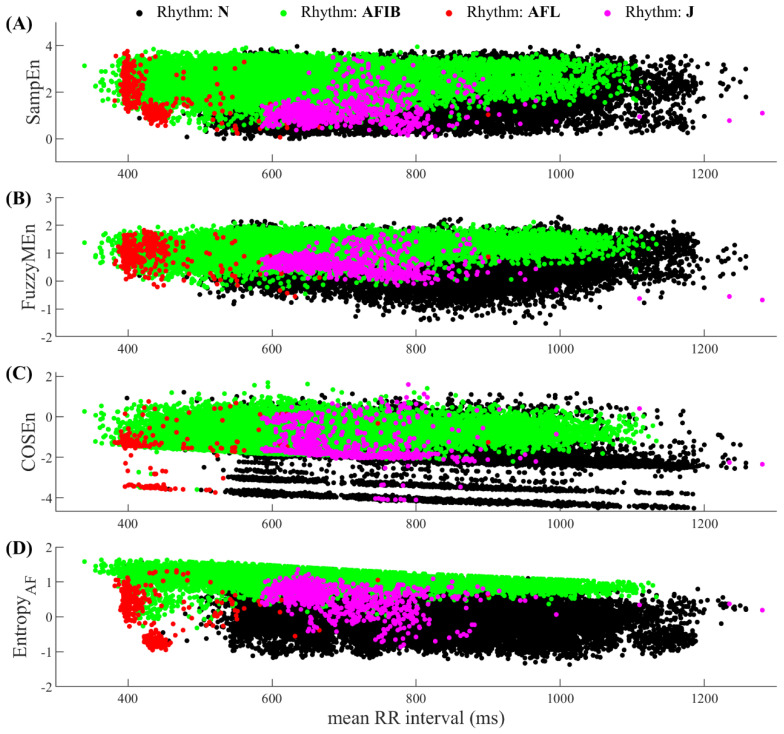
Result distributions of the four entropy measures: (**A**) SampEn, (**B**) FuzzyMEn, (**C**) COSEn and (**D**) *Entropy_AF_* for the four rhythm types (AF, N, AFL and J) in the MIT-BIH AF database. The x-axis is the mean RR interval for the analyzed 30-beat time window.

**Figure 2:**
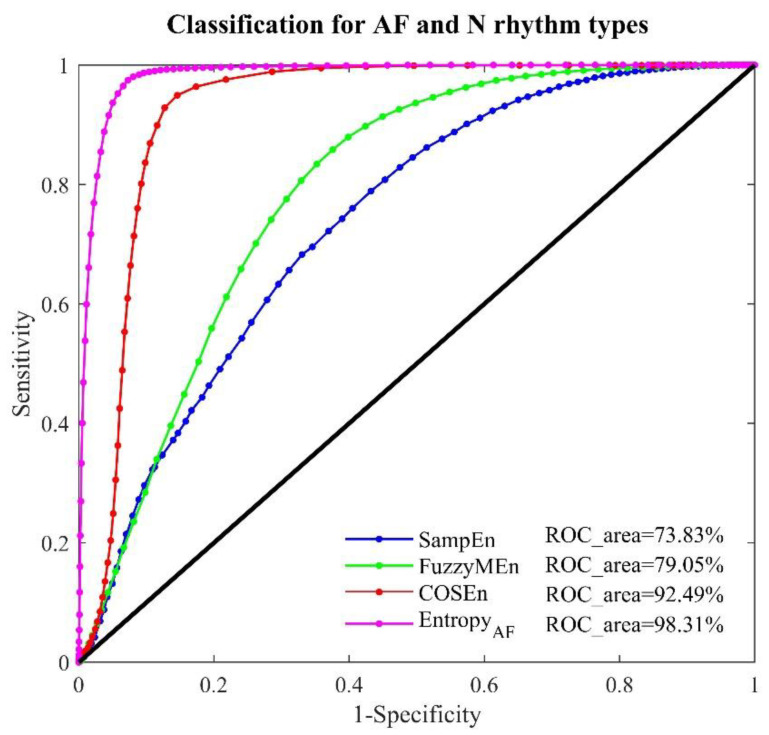
ROC curve plots with AUC values for the four entropy measures: SampEn, FuzzyMEn, COSEn and *Entropy_AF_* in the MIT-BIH AF database for classifying AF and N rhythms using 30-beat RR segments.

Figure 1 shows the distributions of the four entropy measures for AF, N, AFL and J rhythms in the MIT-BIH AF database. It is clear that the departures of AF rhythm from the N rhythm are more obvious in the new proposed *Entropy_AF_* than those in the other three entropy measures. Figure 2 illustrates ROC curves with AUC values for the four entropy measures for classifying AF and N rhythm types on the MIT-BIH AF database. SampEn, FuzzyMEn, COSEn and the new proposed *Entropy_AF_* generate the lowest to highest AUCs, in order, with the values of 73.83%, 79.05%, 92.49% and 98.31% respectively. The larger AUC in *Entropy_AF_* was statistically significant compared with the other three entropy measures (all *p* < 0.01). AUC results for classifying AF and non-AF rhythms show a similar trend as seen in the classification of AF and N rhythms.

**Figure 3:**
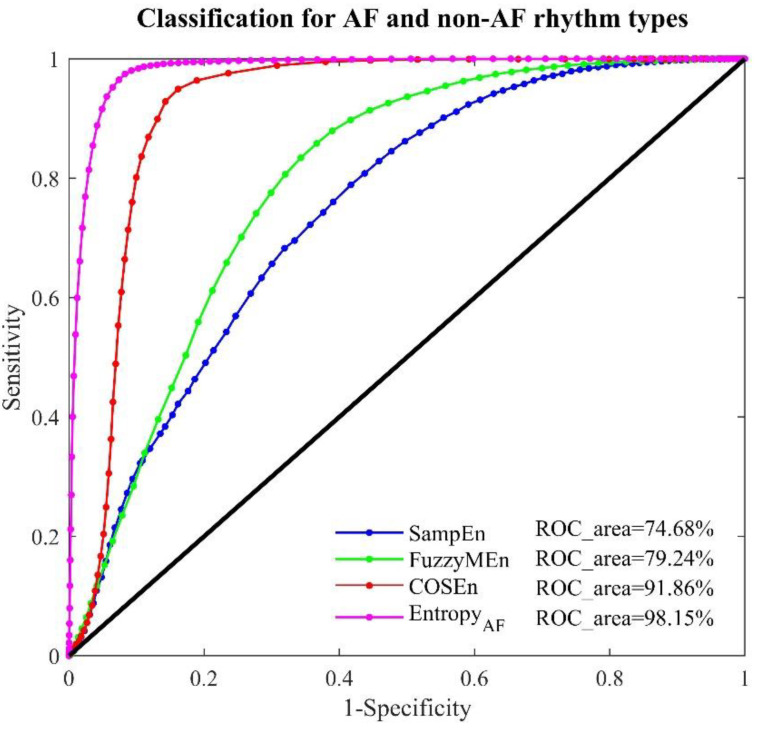
ROC curve plots with AUC values for the four entropy measures: SampEn, FuzzyMEn, COSEn and *Entropy_AF_* in the MIT-BIH AF database for classifying AF and non-AF rhythms using 30-beat RR segments.

Figure 3 illustrates the corresponding ROC curves with AUC values. For classifying AF and non-AF rhythms, SampEn, FuzzyMEn, COSEn and *Entropy_AF_* also result in the lowest to highest AUCs, in order, with the values of 74.68%, 79.24%, 91.86% and 98.15% respectively.

Again, the larger AUC in *Entropy_AF_* was statistically significant (all *p* < 0.01). Because the AFL and J rhythm types are included in the non-AF rhythm, the AUC values for both COSEn and *Entropy_AF_* slightly decreased. AUC values decreased by 0.63% for COSEn and by 0.16% for *Entropy_AF_*.

Table 4 summarizes the classifier performance metrics at the optimal cut-point $c^{*}$, where the classifier equally weighs sensitivity and specificity. Compared with other entropy measures, *Entropy_AF_* generally resulted in the largest values of J, Se, Sp, Acc, PPV and NPV, and the smallest values of Err. Specifically, for classifying AF and N rhythm types, *Entropy_AF_* resulted in the highest Se of 97.45%, Se of 92.71% and Acc of 94.80% respectively. Meanwhile, it also reported the highest PPV of 91.30% and NPV of 97.89%. The Err is only 5.20% for *Entropy_AF_*. COSEn had the second-best performance on the classification of AF and N rhythms, outputting an Acc of 89.73%. FuzzyMEn followed by an Acc of 73.04% and SampEn gave the worst Acc of 67.56%. The optimal cut-point $c^{*}$ values for SampEn, FuzzyMEn, COSEn and *Entropy_AF_* are 2.29, 0.98, −1.51 and 0.72 respectively. As for classifying AF and non-AF rhythm types, similar results were found, at the setting of the optimal cut-point $c^{*}$ values of 2.05, 0.91, −1.58 and 0.76 respectively for the four entropy measures. *Entropy_AF_* generated the highest Acc of 94.25%, followed by an Acc of 88.57% in COSEn, and then followed by an Acc of 72.67% in FuzzyMEn and an Acc of 66.37% in SampEn.

Table 5 summarizes the classifier performance metrics at the cut-point c for highly weighting the sensitivity (Se>99%). Compared with other three entropy measures, *Entropy_AF_* resulted in obviously larger values of Sp of 87.91% and Acc of 92.85% for classifying AF and N rhythms, obviously larger values of Sp of 86.01% and Acc of 91.60% for classifying AF and non-AF rhythms. Acc values for COSEn were 79.69% and 77.99% respectively for the above two classification tasks. As for SampEn and FuzzyMEn, Acc values were even lower than 60%.

Table 6 summarizes the classifier performance metrics at the cut-point c for highly weighting the specificity (Sp>99%). Compared with other three entropy measures, *Entropy_AF_* resulted in an obviously larger value of Se of 53.82% for classifying AF and N rhythm types, an obviously larger values of Se of 53.82% for classifying AF and non-AF rhythm types. Se values for SampEn, FuzzyMEn and COSEn were only 0.91%, 1.22% and 1.90% for classifying AF and N rhythms, and were similarly 0.91%, 1.22% and 1.36% for classifying AF and non-AF rhythms. Acc values in *Entropy_AF_* were 20% larger than those in other three entropy measures.

### 3.2. Results on the Clinical Wearable AF Database (30-Beat Time Window)

Table 7 summarizes the classifier performance metrics for the clinical wearable AF database at the three setting of three cut-point c values: optimal cut-point $c^{*}$ (equally weighs sensitivity and specificity), cut-point c for highly weighting sensitivity (Se>99%) and highly weighting specificity (Sp>99%). For equally weighing sensitivity and specificity, the optimal cut-point $c^{*}$ values of 2.05, 0.91, −1.58 and 0.76 for the four entropy measures obtained from the MIT-BIH AF database analysis were used. *Entropy_AF_* resulted in the largest values of J (77.94%), Se (92.77%), Sp (85.17%), Acc (87.10%), PPV (68.09%) and NPV (97.18%), and the smallest value of Err (12.90%). COSEn had the second-best performance on the classification of AF and non-AF rhythms, outputting an Acc of 78.63%, followed by an Acc of 65.08% for FuzzyMEn and an Acc of 59.91% for SampEn.

For highly weighting the sensitivity (Se>99%), *Entropy_AF_* resulted in obviously larger values of Sp of 73.14% and Acc of 79.73% than other three entropy measures. Acc values for COSEn was 68.54%, and even as low as 40.18% for FuzzyMEn and 34.93% for SampEn. PPV values in this situation were low since many non-AF segments were falsely determined as AF segments due to the low entropy thresholds. SampEn, FuzzyMEn, COSEn and *Entropy_AF_* reported PPV values as 27.99%, 29.72%, 44.66% and 55.72% respectively.

For highly weighting the specificity (Sp>99%), *Entropy_AF_* also resulted in obviously larger values of Se of 40.43% and Acc of 84.11% than the other three entropy measures. Se values for SampEn, FuzzyMEn and COSEn were only 2.67%, 3.54% and 8.11%. PPV values showed a significant increase trend for the four entropy measures, and were 48.06%, 55.00%, 73.78% and 93.35% in turn.

### 3.3. Results When Using a 12-Beat Time Window

**Figure 4:**
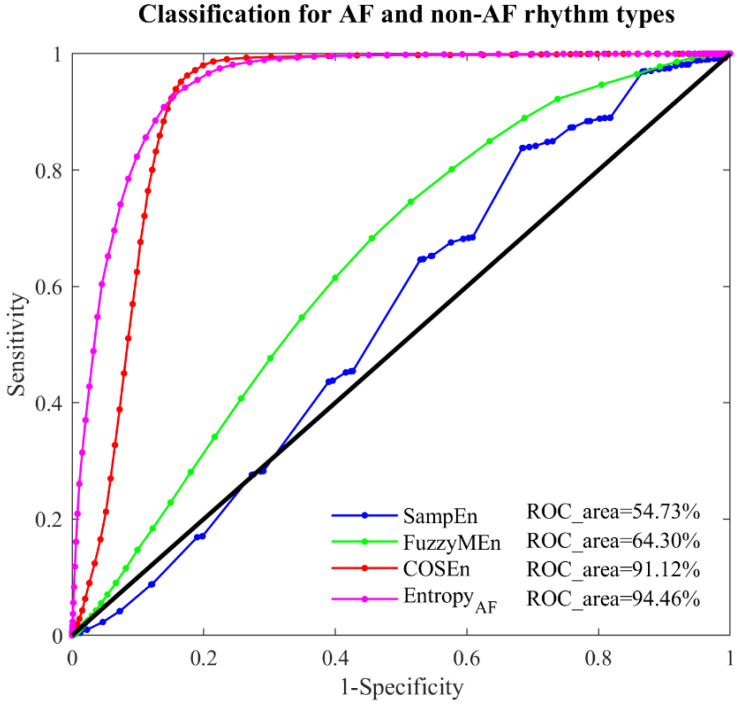
ROC curve plots with AUC values for the four entropy measures: SampEn, FuzzyMEn, COSEn and *Entropy_AF_* in the MIT-BIH AF database for classifying AF and non-AF rhythms using 12-beat RR segments.

Since the performances from classifying AF and N rhythms were similar with those from classifying AF and non-AF rhythms, this section only reported the results for classifying AF and non-AF rhythms when using a 12-beat time window. Figure 4 illustrates the ROC curves with AUC values for the four entropy measures for classifying AF and non-AF rhythms on the MIT-BIH AF database. Compared with the 30-beat time window, the four entropy measures, SampEn, FuzzyMEn, COSEn and *Entropy_AF_*, reported the similar trend with increased classification accuracy, generating the lowest to highest AUCs, in order, with the values of 54.73%, 64.30%, 91.12% and 94.46% respectively. The larger AUC in *Entropy_AF_* was still statistically significant compared with the other three entropy measures (all *p* < 0.01).

Table 8 summarizes the classifier performance metrics for classifying AF and non-AF rhythms when using 12-beat time window. For the MIT-BIH AF database (43,307 AF vs. 58,311 non-AF segments), *Entropy_AF_* generated the largest values of J, Se, Sp, Acc, PPV and NPV, and the smallest values of Err, and reported the highest Acc of 89.01%, followed by an Acc of 88.35% in COSEn, and then followed by an Acc of 59.62% in FuzzyMEn and an Acc of 44.25% in SampEn. For the clinical wearable AF database (14,023 AF vs. 41,055 non-AF segments), *Entropy_AF_* still generated the highest Acc of 79.59%, followed by an Acc of 72.85% in COSEn, and then followed by an Acc of 53.31% in FuzzyMEn and an Acc of 51.31% in SampEn.

### 3.4. Results on the Time Cost

In this study, all of the tests were implemented in MATLAB 2018a (The MathWorks, Inc., Natick, MA, USA) on a 1.80 GHz Intel TM i7-8550U CPU (equipped with 8.00 G RAM). Table 9 illustrates the time costs for the four entropy measures, from analyzing two time windows (30-beat and 12-beat RR segments) on the MIT-BIH AF database. The results were presented as the mean time cost per segment in the unit of ms. All four entropy measures had high numerical efficiency (all <2 ms/segment). When processing a 30-beat RR segment, SampEn had the least mean time cost of 0.24 ms, followed by FuzzyMEn of 0.66 ms. Different from SampEn and FuzzyMEn, which had constant threshold *r* values, COSEn and *Entropy_AF_* needed to optimize the flexible threshold *r* value. Thus, they both reported larger time costs, and had 1.72 ms and 1.62 ms respectively. Compared with COSEn, since *Entropy_AF_* used a normalized vector distance, the optimization process for threshold *r* was straightforward and efficient, generating a relatively lower time cost. When processing a 12-beat RR segment, all four entropy measures needed less time costs, reporting the mean time costs of 0.15 ms, 0.49 ms, 1.58 ms and 1.47 ms respectively.

## 4. Discussion

This study proposed a new entropy-based AF detection method (*Entropy_AF_*), which combined several improvements from the ranged function for similarity determination, coefficient entropy AF detection method and fuzzy set approach. As an application, the new proposed *Entropy_AF_*, as well as three comparable methods (SampEn, FuzzyMEn and COSEn), have been applied to test the discrimination ability between AF and non-AF rhythms. This study showed that, with the new proposed *Entropy_AF_* method, the classification performances between AF and non-AF rhythms were significantly enhanced compared with the three comparable entropy measures. The better discrimination ability of *Entropy_AF_* was confirmed from the analysis of clinical wearable long-term AF recordings. Thus, *Entropy_AF_* analysis holds potential in clinical AF scanning.

Unlike the previous SampEn, FuzzyMEn and COSEn, the unique aspect of the new *Entropy_AF_* is that it used the normalized vector distance definition, enabling it is not sensitive to changes in signal magnitude (gain) [26]. This is useful for studying the dynamics of short-term AF episodes, to accurately and robustly measure the transient changes in RR interval variability. Meanwhile, the *Entropy_AF_* measure is needless of any amplitude correction, which is important for analyzing physiological signals since the physiological signals are usually affected by confounding amplitude changes such as artifacts.

The discrimination ability of *Entropy_AF_* was quantified by the AUC values from ROC curve analysis when performed on the online open-access MIT-BIH AF database, using two time windows (30-beat and 12-beat RR segments). As shown in Figure 2, Figure 3 and Figure 4, its classification performances were consistently better than the comparable methods (with significant statistical differences, all *p* < 0.01), no matter for classifying AF and N rhythms (AUC = 98.31%, 30-beat time window), or for classifying AF and non-AF rhythms (AUC = 98.15%, 30-beat time window, AUC = 94.46%, 12-beat time window). Recently, we have developed a normalized fuzzy entropy (NFEn) for AF detection, which combined the concepts of density estimation, flexible distance threshold and heart rate adjustment, but didn’t employ the concept of ranged function for defining the vector similarity. Results of NFEn on the MIT-BIH AF database reported an AUC of 95.70% (30-beat time window) for AF and N rhythms discrimination, and AUCs of 95.27% (30-beat time window) and 92.72% (12-beat time window) for AF and non-AF rhythms discrimination [12]. Thus, compared with NFEn, the new developed *Entropy_AF_* in this study obtained an AUC increase of 2.61% (30-beat time window) for classifying AF and N rhythms, and an AUC increases of 2.88% (30-beat time window) and 1.74% (12-beat time window) for classifying AF and non-AF rhythms. Meanwhile, compared with COSEn, *Entropy_AF_* obtained a larger AUC increase of 5.82% (98.31% vs. 92.49%) for classifying AF and N rhythms, and a large AUC increase of 6.29% (98.15% vs. 91.86%) for classifying AF and non-AF rhythms (all using 30-beat time window).

The need for improved detection of silent AF is demonstrated by the fact that almost half of the patients with an AF-related stroke, arrhythmia has been found to be asymptomatic and undiagnosed, and therefore untreated [31,32]. Development of a quick, single measure for scanning the onset and offset of AF episodes is a major challenge, which may significantly help on knowing the exact AF duration and calculating the accurate AF burden [33,34]. From the ROC analysis on the MIT-BIH AF database, we concluded the optimal cut-point $c^{*}$ values for SampEn, FuzzyMEn, COSEn and *Entropy_AF_*, and used these cut-point values for AF and non-AF rhythms classification for the clinical wearable AF recordings. The clinical long-term AF data confirmed the fine performance of the new *Entropy_AF_* measure. When tested on 30-beat time window, *Entropy_AF_* generated a high Acc value of 87.10% for classifying AF and non-AF rhythms, while the Acc values were only 59.91%, 65.08% and 78.63% for SampEn, FuzzyMEn and COSEn respectively. Considering that the Acc value of 87.10% was from the clinical wearable long-term ECG recordings that included lots of unexpected and various signal noises, the use of the new *Entropy_AF_* on wearable ECG monitoring will promote a high patient compliance and enhance the utility of the device for AF scanning and postoperative monitoring in individual patients as well as in large population-based AF daily screening.

Furthermore, a varied decision-making threshold for the AF determination can be obtained from the ROC analysis. This would allow the choice of clinically meaningful thresholds since the clinical conditions may differ according to etiology and demographics, such as a cardiac surgery risk threshold may be different than a general Intensive Care Unit (ICU) risk threshold [35]. Herein, we provided two options for the entropy measures: highly weighting the sensitivity (Se>99%) and highly weighting the specificity (Sp>99%), to facilitate the doctors’ judgement for surely accepting AF determination or surely excluding the non-AF determination. The results verified that *Entropy_AF_* can high-accurately detect the AF rhythm (Se>99%), and efficiently identify the non-AF rhythm with a Sp of 73.14%. In contrast, *Entropy_AF_* can also high-accurately exclude the non-AF rhythm (Sp>99%) and pick off about half AF rhythm segments.

The time cost test verified the calculation efficiency of the new *Entropy_AF_* method. All four entropy measures had high numerical efficiency (less than 2 ms) for processing a 30-beat or 12 beat RR segment. SampEn and FuzzyMEn had less time costs since they both used constant threshold *r* values. COSEn and *Entropy_AF_* generated larger time costs since they both needed to optimize the flexible threshold *r*. The interesting fact is that *Entropy_AF_* reported a little smaller mean time cost than COSEn for both 30-beat and 12-beat time windows. The reason should be that *Entropy_AF_* used a normalized vector distance to reduce the complexity of the optimization process for threshold *r*.

The limitations should be summarized. First, the proposed entropy-based AF detection method was a ventricular response analysis-based method, i.e., it only analyzed the irregularity of RR time series within a fixed short time window (30-beat here). Analysis of atrial activity (P wave) can definitely lead to improved performance. Babaeizadeh et al. reported that combining the analysis of RR interval and P wave generated a Se of 93% and Sp of 98% for the MIT-BIH AF database [34], while we got a comparable results of Se as 97.45% and Sp as 92.71%. Combining the P wave analysis will be our near future work, although P wave detection is often difficult when various noises are present in wearable environment. Second, the decrease of performance in the evaluation metrics for clinical wearable ECG signal is mainly due to the false location of QRS complexes in noisy episode. Once error in QRS detection happens, the rhythm will become irregular even if within the non-AF signal episode. In this situation, the rhythm may erroneously be classified as AF. The reduction of the false positives AF is mainly achieved by the improvement of robust QRS location method, which will prevent misclassifying non-AF arrhythmias as AF. Last, we note that employing machine learning methods that combine multiple metrics may improve AF classification performance compared to using a single measure alone [36,37]. We leave these issues to future studies.

In summary, this study demonstrated that compared with the precious three entropy-based AF detectors, the new *Entropy_AF_* has better discrimination ability for the AF and non-AF rhythms. In future experiments, we expect that the newly proposed *Entropy_AF_* analysis will be useful in the practical clinical wearable applications for AF scanning in daily monitoring since it is conceptually simple and computationally efficient.

## Figures and Tables

**Table 1:** MIT-BIH AF database profile separated by the different rhythm types. For each rhythm type, the numbers and the corresponding percentages (%) were given.

Variable	AF Rhythm	Non-AF Rhythm			
		N	AFL	J	Total
# rhythm episodes	299 (48.0%)	292 (46.9%)	14 (2.2%)	18 (2.9%)	324 (52.0%)
Total time length (h)	93.5 (37.5%)	149.1 (59.8%)	1.4 (0.6%)	5.2 (2.1%)	155.7 (62.5%)
# RR intervals	521,415 (42.6%)	663,202 (54.2%)	11,710 (1.0%)	26,818 (2.2%)	701.730 (57.4%)
# RR intervals (≤2 s)	521,359 (42.6%)	662,971 (54.2)	11,710 (1.0%)	26,813 (2.2%)	701,494 (57.4%)
# RR segments	17,247 (42.6%)	21,968 (54.3%)	383 (0.9%)	886 (2.2%)	23,237 (57.4%)

**Table 2:** Signal quality indices (SQIs) used in this study.

SQI	Description	
bSQI [28,29]	Agreement level of two QRS detectors within a fixed time window (10-s).	
tSQI [27]	Morphology consistency of any two ECG beats within a fixed time window (10-s).	
iSQI [27]	Interval abnormal index for RR time series with a fixed time window (10-s).	
pSQI [28,29]	Power spectrum distribution—power ratio between 5–25 Hz and 5–50 Hz.	
kSQI [28,29]	The fourth moment (kurtosis) of the ECG signal distribution.	

**Table 3:** Clinical wearable AF database profile separated by the AF and non-AF rhythm types. For each rhythm type, the numbers and the corresponding percentages (%) were given.

Variable	Noisy Signal	AF Rhythm	Non-AF Rhythm	Total
Total time length (h)	71.8 (31.6%)	35.2 (15.5%)	120.3 (52.9%)	227.3 (100%)
# Valid RR intervals	--	169,741 (25.5%)	495,539 (74.5%)	665,280 (100%)
# RR segments	--	5587 (25.4%)	16,376 (74.6%)	21,963 (100%)

**Table 4:** Results of the performance metrics at the setting of the optimal cut-point $c^{*}$ for the four entropy measures in the MIT-BIH AF database.

Task	Metric	SampEn	FuzzyMEn	COSEn	Entropy_AF_
AF vs. N rhythms	$c^{*}$	2.29	0.98	−1.51	0.72
	J(%)	34.53	47.71	80.12	90.17
	Se(%)	65.64	80.65	92.83	97.45
	Sp(%)	68.88	67.07	87.28	92.71
	Acc(%)	67.56	73.04	89.73	94.80
	PPV(%)	59.16	65.78	85.14	91.30
	NPV(%)	74.49	81.53	93.94	97.89
	Err(%)	32.44	26.96	10.28	5.20
AF vs. non-AF rhythms	$c^{*}$	2.05	0.91	−1.58	0.76
	J(%)	37.16	48.73	78.78	89.07
	Se(%)	78.88	85.82	94.93	96.47
	Sp(%)	58.28	62.91	83.85	92.59
	Acc(%)	66.37	72.67	88.57	94.25
	PPV(%)	55.03	63.20	81.35	90.63
	NPV(%)	81.00	85.67	95.70	97.25
	Err(%)	33.63	27.33	11.43	5.75

**Table 5:** Results of the performance metrics at the setting of the cut-point c for highly weighting the sensitivity (Se>99%) for the four entropy measures in the MIT-BIH AF database.

Task	Metric	SampEn	FuzzyMEn	COSEn	Entropy_AF_
AF vs. N rhythms	c	1.04	0.29	−1.88	0.54
	J(%)	15.71	24.42	63.64	87.05
	Se(%)	>99.0	>99.0	>99.0	>99.0
	Sp(%)	16.64	25.34	64.14	87.91
	Acc(%)	50.20	57.77	79.69	92.85
	PPV(%)	44.94	51.03	68.54	86.55
	NPV(%)	96.32	97.24	99.39	99.24
	Err(%)	49.80	42.23	20.31	7.15
AF vs. non-AF rhythms	c	1.04	0.29	−1.88	0.54
	J(%)	16.89	23.63	61.53	85.15
	Se(%)	>99.0	>99.0	>99.0	>99.0
	Sp(%)	17.82	24.55	62.03	86.01
	Acc(%)	49.75	56.30	77.99	91.60
	PPV(%)	43.83	49.36	66.04	84.02
	NPV(%)	96.74	97.30	99.41	99.26
	Err(%)	50.25	43.70	22.01	8.40

**Table 6:** Results of the performance metrics at the setting of the cut-point c for highly weighting the sensitivity (Sp>99%) for the four entropy measures in the MIT-BIH AF database.

Task	Metric	SampEn	FuzzyMEn	COSEn	Entropy_AF_
AF vs. N rhythms	c	3.53	1.79	0.35	1.09
	J(%)	0.12	0.41	0.93	52.87
	Se(%)	0.91	1.22	1.90	53.82
	Sp(%)	>99.0	>99.0	>99.0	>99.0
	Acc(%)	59.19	56.10	56.31	79.16
	PPV(%)	44.06	54.12	60.52	97.81
	NPV(%)	59.31	56.12	56.25	73.20
	Err(%)	40.81	43.90	43.69	20.84
AF vs. non-AF rhythms	c	3.53	1.79	0.43	1.09
	J(%)	0.13	0.44	0.57	52.83
	Se(%)	0.91	1.22	1.36	53.82
	Sp(%)	>99.0	>99.0	>99.0	>99.0
	Acc(%)	60.59	57.47	57.53	79.76
	PPV(%)	43.15	53.85	56.25	97.59
	NPV(%)	60.74	57.51	57.54	74.28
	Err(%)	39.41	42.53	42.47	20.24

**Table 7:** Results of the performance metrics for the four entropy measures in the clinical wearable AF database.

Metric	SampEn	FuzzyMEn	COSEn	*Entropy_AF_*
$c^{*}$	2.05	0.91	−1.58	0.76
J(%)	33.54	42.32	64.50	77.94
Se(%)	80.74	83.53	89.62	92.77
Sp(%)	52.80	58.79	74.88	85.17
Acc(%)	59.91	65.08	78.63	87.10
PPV(%)	36.85	40.88	54.90	68.09
NPV(%)	88.93	91.28	95.48	97.18
Err(%)	40.09	34.92	21.37	12.90
c	1.04	0.29	−1.88	0.54
J(%)	12.11	19.13	57.17	72.19
Se(%)	>99.0	>99.0	>99.0	>99.0
Sp(%)	13.06	20.10	58.14	73.14
Acc(%)	34.93	40.18	68.54	79.73
PPV(%)	27.99	29.72	44.66	55.72
NPV(%)	97.58	98.39	99.44	99.56
Err(%)	65.07	59.82	31.46	20.27
c	3.53	1.79	0.43	1.09
J(%)	1.68	2.55	7.12	39.45
Se(%)	2.67	3.54	8.11	40.43
Sp(%)	>99.0	>99.0	>99.0	>99.0
Acc(%)	74.51	74.73	75.89	84.11
PPV(%)	48.06	55.00	73.78	93.35
NPV(%)	74.89	75.05	75.95	82.97
Err(%)	25.49	25.27	24.11	15.89

**Table 8:** Results of the performance metrics for the four entropy measures when using a 12-beat time window.

Database	Metric	SampEn	FuzzyMEn	COSEn	*Entropy_AF_*
	$c^{*}$	1.08	1.01	−1.32	0.04
MIT-BIH AF database	J(%)	15.38	23.08	78.74	78.80
	Se(%)	83.80	74.54	96.24	94.17
	Sp(%)	31.58	48.63	82.49	84.63
	Acc(%)	44.25	59.62	88.35	89.01
	PPV(%)	28.19	51.82	80.33	82.34
	NPV(%)	85.89	71.97	96.73	94.17
	Err(%)	55.75	40.38	11.65	11.26
Clinical wearable AF database	J(%)	15.05	19.79	53.18	63.00
	Se(%)	70.18	73.32	84.20	85.38
	Sp(%)	44.87	46.47	68.98	77.62
	Acc(%)	51.31	53.31	72.85	79.59
	PPV(%)	30.30	31.87	48.11	56.58
	NPV(%)	81.50	83.61	92.74	93.96
	Err(%)	48.69	46.69	27.15	20.41

**Table 9:** Results of mean time cost for the four entropy measures when performing on 30-beat and 12-beat RR segments in the MIT-BIH AF database.

Time Window	# Total Segments	Mean Time Cost (Unit: ms/Segment)			
		SampEn	FuzzyMEn	COSEn	*Entropy_AF_*
30-beat	40,484	0.24	0.66	1.72	1.62
12-beat	101,618	0.15	0.49	1.58	1.47
